# Inhibition of emotional needs and emotional wellbeing predict disease progression of chronic hepatitis C patients: an 8-year prospective study

**DOI:** 10.1186/s13030-016-0075-3

**Published:** 2016-07-29

**Authors:** Ryoko Sawamoto, Jun Nagano, Eiji Kajiwara, Junko Sonoda, Tetsuya Hiramoto, Nobuyuki Sudo

**Affiliations:** 1 Department of Psychosomatic Medicine, Graduate School of Medical Sciences, Kyushu University, 3-1-1 Maidashi, Higashi-ku Fukuoka, 812-8582 Japan; 2 Institute of Health Science, Kyushu University, Fukuoka, Japan; 3 Steel Memorial Yawata Hospital, Kitakyushu, Japan

**Keywords:** Chronic hepatitis, Psychosocial stress, Quality of life, Emotion, Hepatocellular carcinoma, Prognosis

## Abstract

**Background:**

The role of psycosocial factors in the disease progression of chronic hepatitis C (CHC) patients remains unclear. The aim of the present study was to prospectively evaluate the prognostic value of behavioral patterns and the quality of life (QOL) of patients with CHC.

**Methods:**

Two hundred and forty Japanese CHC patients (mean age 62.4 years) were assessed for behavioral patterns (Stress Inventory), QOL (Functional Assessment of Chronic Illness Therapy-Spiritual), and known prognostic factors at baseline then followed for a maximum of 8 years for disease progression, defined as either the first diagnosis of hepatocellular carcinoma (HCC) or hepatitis-related death.

**Results:**

Forty-nine events occurred during the study period (46 newly diagnosed HCC cases, three hepatitis-related deaths). In a Cox proportional hazard model including known prognostic factors and treatment-related factors as time-dependent variables, behavioral patterns associated with inhibition of emotional needs (hazard ratio (HR): 1.35; 95 % confidence interval (CI): 1.02–1.77; *p* = 0.036) and QOL, representing emotional wellbeing (HR 0.60; 95 % CI 0.37–0.98; *p* = 0.041), were each associated with the risk of disease progression.

**Conclusion:**

Psychosocial factors such as behavioral patterns relevant to the inhibition of emotional needs and emotional wellbeing independently affect the clinical course of patients with CHC.

**Electronic supplementary material:**

The online version of this article (doi:10.1186/s13030-016-0075-3) contains supplementary material, which is available to authorized users.

## Background

Chronic hepatitis C (CHC) is a major public health problem. According to recent estimates, more than 130-150 million people around the world have been infected with the hepatitis C virus (HCV). Of those with chronic HCV infection, the risk of cirrhosis is 15–30 % within 20 years. A significant number of those who are chronically infected will deverop hepatocellular carcinoma (HCC) which is a major life-threatening complication. Approximately 700,000 people die each year from hepatitis C-related liver diseases.[1]. Extant research has found that HCV genotype 1b [2], older age, male sex, elevation in the serum bilirubin, decreased platelet count, and alcohol intake [3] contribute to the development of HCC by patients with CHC; however, the role of psychosocial factors in the initiation and progression of HCC remains unclear.

In a series of animal experiments, we demonstrated a relationship between stress and liver diseases [4–6]. For example, electric foot-shock stress exacerbated liver injury in a hepatitis model using α-galactosylceramide [4]. In a Fas-induced liver injury model, we showed that the hepatic sympathetic nerve protects against Fas-induced liver injury by maintaining interleukin-6 levels in the liver [5]. Furthermore, in a study using a selective hepatic vagotomy technique, the hepatic vagus nerve attenuated Fas-induced hepatocyte apoptosis through the α7 nicotinic acetylcholine receptor [6]. These findings collectively suggest that the liver reciprocally interacts with the brain via the endocrine and autonomic nerve systems. In accordance with these findings obtained from animal studies, some decades-old clinical literature suggests that psychosocial stress may affect the initiation and clinical course of liver diseases. For example, Fukudo et al. found that the severity of psychosocial stress was significantly correlated with the exaggeration of inflammatory and fibrosing changes in patients with alcoholic hepatitis [7]. Moreover, we previously reported that the “type-I behavior pattern” was associated with CHC severity after adjusting for age, sex, education level, smoking, drinking, and duration of illness [8]. Type-I behavior, originally proposed by Grossarth-Maticek as a cancer-prone behavioral pattern [9, 10], is characterized by marked repression of emotions and altruistic behavior in interpersonal relationships [9]. Nonetheless, as the observed association between type-I behavior and CHC severity was based on retrospective data, the causal relationship between such psychosocial factors and the clinical course of CHC remains undetermined.

Recently, research has examined quality of life (QOL) in terms of its ability to predict the clinical course of chronic diseases, including various types of cancer [11, 12] (e.g. of the lung [13, 14], esophagus [15], head and neck [16], colon [17], and ovary [18]), in addition to its role as an outcome of chronic diseases (e.g. the negative effect of CHC on patients’ QOL [19]). Further, QOL may predict the clinical course of patients with HCC [20–22]. Nonetheless, there is presently no prospective data on QOL as a predictor of the course of CHC. The present study therefore aimed to evaluate the prognostic values of psychosocial factors in a group of patients with CHC, based on 8 years of prospective data that incorporated known prognostic factors.

## Methods

### Enrollment of subjects

The study protocol was approved by the Ethics Committee of the Faculty of Medical Science, Kyushu University. Patients with CHC who were regularly visiting the Steel Memorial Yawata Hospital in Kitakyushu city, Japan during the period from September to December 2003 were invited to participate. Criteria for recruitment were 1) age under 80 years; 2) no decompensated cirrhosis symptoms, such as intractable ascites, variceal bleeding, or encephalopathy; 3) no history of malignant diseases including HCC; 4) no medical conditions preventing completion of a self-administered questionnaire without assistance; and 5) no co-infection with hepatitis B virus. Patients who had succeeded in eliminating hepatitis C virus with interferon treatment and continued visiting the hospital for follow-up were also included.

The attending hepatologist informed a series of eligible patients of the study’s details and asked the patients to participate. Participating patients signed a consent form, completed a set of questionnaires at home, and mailed them to a central office (Faculty of Psychosomatic Medicine, Kyushu University Graduate School of Medical Sciences). A gift certificate worth around 10 USD was then sent to the participants as gratitude.

### Psychosocial assessment

#### The stress inventory

The Stress Inventory (SI) is a 45-item, self-report questionnaire developed by Nagano et al. to assess behavioral patterns that hypothetically relate to the development of chronic diseases such as cancer and cardiovascular disease [8, 23, 24]. The SI consists of 12 scales, four of which were designed to measure elements of type-I behavior, which is characterized by marked inhibition of emotional needs [25]: “Low sense of control,” diminished sense of control over stressful situations, leading to hardship, despair, or anger; “object dependence of loss,” having an important person in one’s life who causes persistent hopelessness and depression; “unfulfilled need for acceptance,” chronically having an unfulfilled need for acceptance by others; and “altruism,” an altruistic tendency, accompanied by stress, in interpersonal and social relationships. Responses used a 6-point scale (1 = *yes* or *almost always*, 6 = *no* or *rarely*); item scores were averaged to give the scale score; higher scale scores thus represented a stronger type-I tendency. The “type-I score” was also calculated by averaging the four scale scores [8].

#### The Functional Assessment of Chronic Illness Therapy-Spiritual (FACIT-Sp)

The FACIT-Sp is a 41-item, self-report questionnaire developed by Cella et al. [26] to assess multiple domains of QOL, including spirituality, and intended for use in research examining people with chronic and/or life-threatening illnesses. It consists of five scales that correspond to five domains of QOL: “physical wellbeing,” comprising reports of physical symptoms; “functional wellbeing,” assessing the degree to which a respondent is able to participate in and enjoy normal daily activities; “social/familial wellbeing,” representing social support and communication; “emotional wellbeing,” measuring mood and emotional response to illness; and “spiritual wellbeing,” representing a sense of meaning in one’s life, inner harmony, peacefulness, and a sense of comfort and strength in one’s spiritual beliefs. Answers are rated on a 5-point Likert scale (0 = *not at all*, 4 = *very much*). Item scores in each scale are averaged to give scale scores; higher scores thus represent greater wellbeing [27]. We used a Japanese version of the FACIT-Sp, the reliability and validity of which have been supported by Noguchi et al. [28].

### Clinical data at baseline and during follow-up

The following known clinical prognostic factors were considered in the present study: age, sex, disease duration, cirrhosis, diabetes mellitus, current and past consumption of alcohol, alpha-fetoprotein (AFP), alanine transaminase (ALT), platelet count, HCV genotype, sustained virological response (SVR) and sustained biolochemical response (SBR) to antiviral therapy (see Additional file 1 regarding assessment of biochemical measurements). SVR was defined as serum HCV RNA continuously negative for >6 months after completion of interferon therapy. SBR was defined as serum ALT decreased into the normal range continuously for >6 months after termination of interferon therapy. Liver cirrhosis was diagnosed using ultrasonography (US), computed tomography (CT), or liver biopsy. HCC was diagnosed based on typical findings on dynamic CT or magnetic resonance imaging, and/or by histology.

### Follow-up

Follow-up commenced on the date when the central office received signed consent form from the participant. Information about the clinical course, such as occurrence of HCC, liver function, and treatment of CHC, were collected by reviewing the participants’ medical charts in September 2011. ALT as a marker of liver injury was measured at each visit, with 1–3 month intervals; however, one record per each follow-up year was abstracted for the present analysis. If a participant did not visit the hospital for more than six months, the attending hepatologist contacted that participant to obtain information about the participants’ medical condition and reasons for the interruption. The hepatologist also contacted the patient’s current attending physician to obtain more detailed information, as appropriate. Follow-up continued until the date of a “disease progression,” until the last visiting date in the case of participant dropout, or until 8 years of continuous follow-up, whichever was earliest. Disease progression was defined as either the first diagnosis of HCC or hepatitis-related death, such as by hepatic failure or upper gastro-intestinal bleeding.

### Statistical analysis

Survival analysis using the Cox proportional hazards model was applied to time-to-event data, where survival was from the start-of-follow-up date to the end-of-follow-up date, and the event was disease progression. Association between each of the psychosocial factors and disease progression was examined using a three-step multivariate Cox regression analysis. Models in the first step included a psychosocial variable of interest (continuous), and age (continuous) and sex (male = 0, female = 1) as covariates. Models in the second step were adjusted for age, sex, baseline known risk factors (i.e. cirrhosis (dichotomous), platelet count (≥ or <100,000/mm^3^), baseline ALT (< or ≥40 IU/L), AFP (< or ≥20 μg/L), diabetes (dichotomous), current consumption of alcohol (dichotomous), and past consumption of alcohol (dichotomous)), where platelet count, baseline ALT, and AFP were averages of the three latest measurements. Models in the third step included these variables and treatment-related factors during the follow-up period as covariates (i.e. ALT and response to antiviral therapy). These treatment-related factors were set as time-dependent variables, where ALT (< or ≥40 IU/L), SVR (dichotomous), SBR (dichotomous), and no response (dichotomous) was the latest value or status at every time-point when an event occurred.

Baseline measurements of psychosocial variables may have been affected by impaired physical conditions, especially for patients who were ill because of hepatitis-related complications including latent HCC, which could not have been represented by the baseline covariates considered. Such an effect might have confounded the association between psychosocial factors and disease progression. To minimize the potential for such confounding, we repeated the above analyses after excluding subjects for whom an event occurred in the first year of follow-up (See previous reports that used similar methods [29–31]). Reported *P*-values were two-sided; SAS 9.4 was used for all statistical analyses.

## Results

**Fig. 1:**
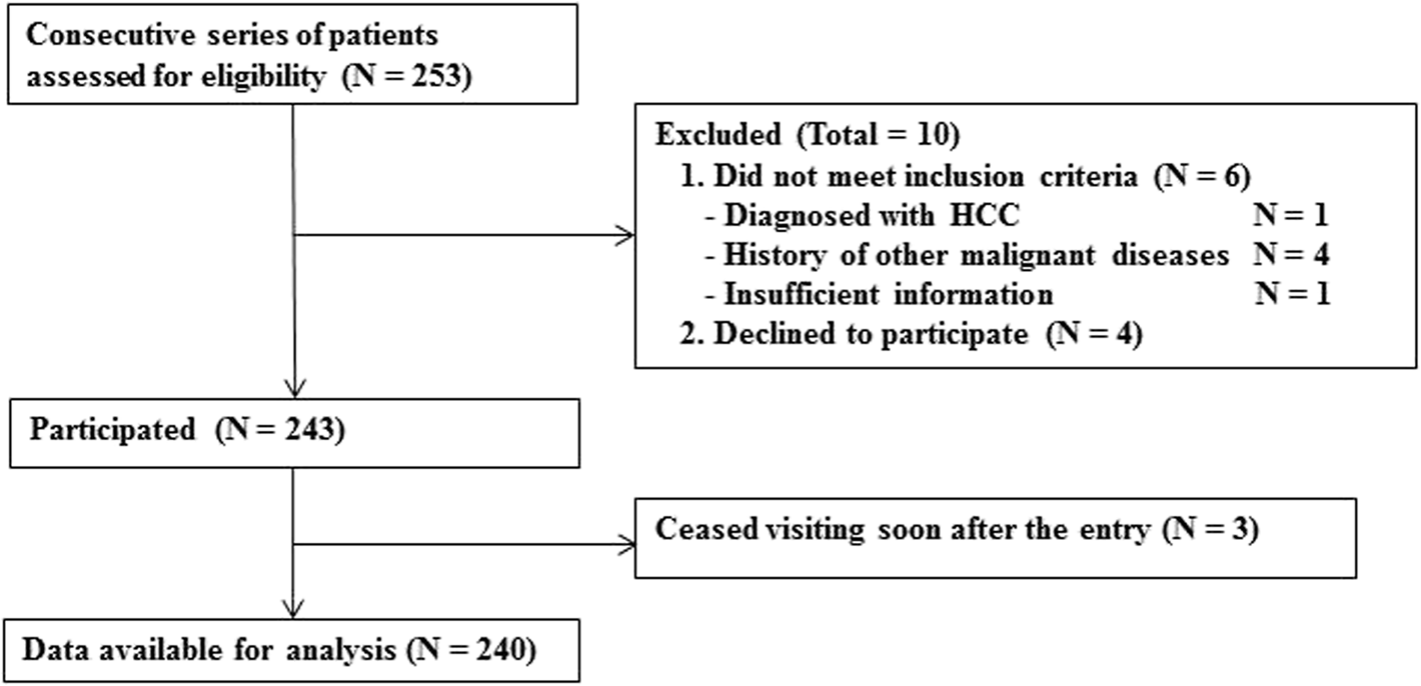
Flow diagram of participants

A consecutive series of 253 patients were assessed for eligibility (see Fig. 1), of whom ten did not meet the eligibility criteria: One was diagnosed with HCC and four with other malignant diseases; four refused to participate; and one had insufficient information for enrollment. Three patients ceased visiting soon after commencing participation and could not be contacted any further. Data for the remaining 240 patients were available for analysis, none of whom had a psychiatric diagnosis or took any psychotropic agents regularly, except for a short-acting sleep inducer. Table 1 summarizes the participants’ baseline characteristics.

**Table 1:** Baseline characteristics of 240 patients with chronic hepatitis C

Characteristic	
Age, years	62.4 (9.1)^a^
Female	140 (58.3 %)
Duration, years	7.7 (5.1)^a^
HCV Genotype 1b	177 (73.8 %)
Anti-viral therapy	93 (38.8 %)
Cirrhosis	54 (22.5 %)
Diabetes	29 (12.1 %)
Current alcohol-drinker	66 (27.5 %)
Past alcohol-drinker	65 (27.1 %)
Aspartate aminotransferase^c^ (IU/L)	40.0 [30.0–56.0]^b^
Alanine aminotransferase^c^ (IU/L)	44.0 [32.0–59.0]^b^
Platelet count (×10^4^/mm^3^)	14.8 [11.0–19.1]^b^
Albumin (g/dl)	4.2 [4.0–4.4]^b^
Bilirubin (mg/dl)	0.9 [0.7–1.1]^b^
Total cholesterol (mg/dl)	172 [153–197]^b^
Alpha fetoprotein (ng/ml)	5.5 [3.8–10.0]^b^

Readings are N (%) unless otherwise specified. ^a^Mean (standard deviation). ^b^Median [inter-quartile range]. ^c^Mean of recent three measurements

During the study period, 49 events occurred, including 46 newly diagnosed HCC and three hepatitis-related deaths (two liver failures; one upper gastro-intestinal bleeding). The remaining 191 were censored because of hepatitis-unrelated death (*N* = 8: pancreas cancer, 2; lung cancer, 1; breast cancer, 1; uterine corpus cancer, 1; bile duct cancer, 1; cerebral bleeding, 1; unknown reason, 1); cessation of visiting (*N* = 69: reasons unrelated to hepatitis, e.g. moving associated with job or family, 24; reasons possibly related to hepatitis, e.g. a complete cure or remission of hepatitis or onset of cerebral bleeding, 25; unknown reasons, 20); or the last visit by patients who continued visiting until 30 September 2011 without an event or death (*N* = 114). Median, range, and inter-quartile range of survival time were 7.5, 0.3–8.0, and 6.7–7.7 years, respectively. A total of 146 patients (60.8 %) received antiviral therapy (either IFN monotherapy or combination therapy with pegylated IFN or IFN with ribavirin) of whom 67, 33, and 46 exhibited SVR, SBR, and no response to therapy at the end of follow-up, respectively. Of the 146 patients, 25 had received antiviral therapy before the start of follow-up, but did not receive further antiviral therapy during the follow-up period. The remaining 121 patients received 173 antiviral therapies in total during the follow-up period: the number of first, second, third, fourth, fifth, and sixth therapies were 90, 46, 24, 9, 3, and 1, respectively; and the number of SVR, SBR, and no response were 66, 23, and 32, respectively.

Table 2 shows the association of known physical and treatment-related risk factors and CHC progression, after controlling for each other factor using multivariate Cox regression models. In Model 1, which included age, sex, and baseline status of known risk factors, only cirrhosis and AFP were significantly and independently associated with CHC progression. When treatment related-factors during the follow-up period were additionally entered as time-dependent variables (Model 2), cirrhosis, AFP, and a recent high value of ALT were significantly associated with subsequent disease progression. A model that only included the time-dependent variables for antiviral therapy estimated the hazard ratios (HRs) of SVR, SBR, and no response to be 0.91 (95 % confidence interval, 0.35–2.32; *P* = 0.84), 1.68 (0.73–3.86; *P* = 0.22), and 2.10 (1.07–4.12; *P* = 0.030), respectively, as compared to no antiviral therapy (not shown in Table 2). As shown in Table 2 (Model 2), however, none of the treatment categories was significantly associated with disease progression, after controlling for the baseline risk factors and a recent high ALT value.

**Table 2:** Multivariate analysis^a^ of demographic and known risk factors in association with subsequent disease progression^b^ in 240 patients with chronic hepatitis C

	Model 1^c^		Model 2^d^	
Factor	HR (95 % CI)	*P*-value	HR (95 % CI)	*P*-value
Age, by 10 years	1.31 (0.88–1.96)	0.18	1.39 (0.93–2.09)	0.11
Female sex	1.10 (0.56–2.19)	0.78	1.28 (0.64–2.57)	0.48
Cirrhosis	4.23 (2.15–8.32)	<0.001	4.36 (2.16–8.79)	<0.001
Platelet count <100,000/mm^3^	1.41 (0.72–2.76)	0.31	1.57 (0.80–3.08)	0.19
Alanine aminotransferase >= 40 IU/l (baseline value)	1.02 (0.55–1.90)	0.94	0.84 (0.43–1.62)	0.60
Alpha fetoprotein >= 20 μg/l	2.50 (1.26–4.95)	0.009	2.35 (1.12–4.91)	0.023
Diabetes	1.17 (0.56–2.44)	0.68	1.14 (0.54–2.38)	0.73
Alcohol-drinking, current^e^	1.00 (0.42–2.41)	1.00	1.08 (0.45–2.60)	0.87
Alcohol-drinking, past^e^	1.56 (0.73–3.35)	0.25	1.42 (0.64–3.15)	0.39
Alanine aminotransferase >= 40 IU/l (recent value) ^f^			2.21 (1.13–4.33)	0.021
Sustained virological response to antiviral therapy^g^			1.66 (0.60–4.60)	0.33
Sustained biological response to antiviral therapy^g^			0.94 (0.38–2.31)	0.89
No response to antiviral therapy^g^			1.17 (0.55–2.49)	0.68

*HR* hazard ratio, *CI* confidence interval. ^a^Using Cox proportional hazards models based on time-to-event data where event was defined as death associated with hepatitis or diagnosis of HCC. ^b^Disease progression was defined as either the first diagnosis of HCC or hepatitis-related death, such as hepatic failure and upper gastro-intestinal bleeding. ^c^Demographic and known risk factors for HCC at baseline. ^d^Demographic and baseline risk factors, and treatment-related factors during the follow-up period. ^e^No alcohol-drinking as reference. ^f^Time-dependent variable, based on a latest measurement at every time-point when an event occurred. ^g^Time-dependent variable, no antiviral therapy as reference

Table 3 shows the association between psychosocial factors and the progression of CHC in the entire sample. In age- and sex-adjusted models, higher scores on the SI Type-I related scales, except for “low sense of control,” were weakly associated with disease progression; however, none of these associations was statistically significant. Additional adjustment for baseline known risk factors did not change these associations, nor did further adjustment for treatment-related factors. Regarding QOL, in age- and sex-adjusted models, FACIT-G total scores and scores on the FACIT-G subscales “physical wellbeing,” “emotional wellbeing,” and “functional wellbeing,” FACIT-Sp total score, scores on the FACIT-Sp “meaning/peace” subscale, and FACIT total score were each significantly inversely associated with risk of disease progression. All of these associations, however, were appreciably attenuated to non-significant levels after additionally controlling for baseline levels of known risk factors and further controlling for treatment-related factors.

**Table 3:** Psychosocial factors in association with subsequent disease progression^a^ in 240 patients with chronic hepatitis C^b^

	Model 1^c^		Model 2^d^		Model 3^e^	
Scale	HR (95 % CI) ^f^	*P* value	HR (95 % CI)	*P* value	HR (95 % CI)	*P* value
Stress Inventory						
Type-I-related scales						
Low sense of control	0.99 (0.78–1.25)	0.92	1.03 (0.80–1.32)	0.83	1.02 (0.79–1.32)	0.88
Object dependence of loss	1.15 (0.87–1.52)	0.32	1.16 (0.87–1.56)	0.31	1.18 (0.87–1.61)	0.28
Unfulfilled need for acceptance	1.19 (0.96–1.49)	0.12	1.17 (0.91–1.51)	0.23	1.18 (0.92–1.53)	0.20
Altruism	1.10 (0.85–1.42)	0.48	1.16 (0.87–1.56)	0.31	1.11 (0.83–1.48)	0.49
Total score	1.17 (0.86–1.59)	0.32	1.21 (0.85–1.70)	0.29	1.19 (0.84–1.69)	0.32
FACIT						
FACIT-G						
Physical wellbeing	0.56 (0.38–0.81)	0.002	0.84 (0.57–1.26)	0.41	0.90 (0.60–1.35)	0.60
Emotional wellbeing	0.64 (0.42–0.97)	0.036	0.73 (0.47–1.14)	0.16	0.73 (0.46–1.16)	0.19
Functional wellbeing	0.69 (0.52–0.91)	0.009	0.79 (0.56–1.13)	0.20	0.80 (0.56–1.13)	0.20
Social/familial wellbeing	1.14 (0.83–1.58)	0.41	1.02 (0.71–1.45)	0.93	0.91 (0.64–1.30)	0.61
Total score	0.55 (0.32–0.93)	0.025	0.69 (0.38–1.27)	0.24	0.67 (0.37–1.22)	0.19
FACIT-Sp						
Meaning/peace	0.65 (0.45–0.94)	0.021	0.82 (0.56–1.21)	0.31	0.73 (0.49–1.08)	0.11
Faith	0.88 (0.65–1.19)	0.40	0.85 (0.60–1.19)	0.33	0.76 (0.53–1.07)	0.11
Total score	0.68 (0.46–0.99)	0.046	0.80 (0.53–1.20)	0.27	0.69 (0.46–1.05)	0.08
Total score (G + Sp)	0.54 (0.32–0.90)	0.019	0.68 (0.38–1.23)	0.21	0.62 (0.35–1.11)	0.11

*HR* hazard ratio, *CI* confidence interval, *FACIT* functional assessment of cancer therapy, *FACIT-G* FACIT-General, *FACIT-Sp*: FACIT-Spiritual

^a^Disease progression was defined as either the first diagnosis of HCC or hepatitis-related death, such as hepatic failure and upper gastro-intestinal bleeding. ^b^Using Cox proportional hazards models fitted to time-to-event data where event was either death associated with hepatitis or diagnosis of hepatocellular carcinoma. ^c^Adjusted for age and sex. ^d^Adjusted for age, sex, and baseline known risk factors; cirrhosis, alanine. ^e^Adjusted for age, sex, baseline known risk factors, and treatment-related factors during the follow-up period; ALT (most recent value) and results of antiviral treatments (sustained virological response, sustained biological response, or no response) as time-dependent variables. ^f^HR associated with a 1-point increment in the scores of the Stress Inventory scales and the FACIT scales

Table 4 shows associations between psychosocial factors and disease progression in the subset of 227 participants in which seven participants diagnosed with HCC and six censored in the first year of follow-up were excluded from the participant set. In age- and sex-adjusted models, associations with scores on the type-I related scales were stronger in this subset than in the complete participant group; however, only the association with “unfulfilled need for acceptance” was statistically significant. These results were similar after controlling for other factors. Age- and sex-adjusted models also yielded stronger relationships for scores on the FACIT scales in the subset than among all participants, except for “social/family wellbeing” (from the FACIT-G) and “faith” (from the FACIT-Sp), which were not related to disease progression. Nonetheless, when the levels of known baseline risk factors and/or treatment-related factors were additionally controlled for, these associations generally weakened to a non-significant level, with only the association with “emotional wellbeing” consistently remaining significant.

**Table 4:** Psychosocial factors in association with subsequent disease progression^a^ in 227 patients with chronic hepatitis C, after excluding patients who met an event or was censored within the first one year of follow-up^b^

	Model 1^c^		Model 2^d^		Model 3^e^	
Scale	HR (95 % CI) ^f^	*P* value	HR (95 % CI)	*P* value	HR (95 % CI)	*P* value
Stress Inventory						
Type-I-related scales						
Low sense of control	1.08 (0.84–1.40)	0.55	1.13 (0.85–1.49)	0.40	1.12 (0.84–1.48)	0.45
Object dependence of loss	1.21 (0.90–1.62)	0.20	1.23 (0.89–1.69)	0.21	1.25 (0.90–2.28)	0.11
Unfulfilled need for acceptance	1.31 (1.03–1.67)	0.026	1.33 (1.01–1.74)	0.041	1.35 (1.02–1.77)	0.036
Altruism	1.16 (0.88–1.53)	0.31	1.24 (0.90–1.70)	0.18	1.19 (0.87–1.63)	0.27
Total score	1.32 (0.94–1.83)	0.11	1.39 (0.96–2.02)	0.08	1.38 (0.95–2.01)	0.10
FACIT						
FACIT-G						
Physical wellbeing	0.53 (0.35–0.78)	0.002	0.76 (0.49–1.18)	0.22	0.80 (0.51–1.24)	0.32
Emotional wellbeing	0.54 (0.34–0.84)	0.006	0.60 (0.37–0.97)	0.036	0.60 (0.37–0.98)	0.041
Functional wellbeing	0.63 (0.47–0.85)	0.002	0.72 (0.49–1.04)	0.08	0.71 (0.49–1.04)	0.07
Social/familial wellbeing	1.11 (0.78–1.56)	0.57	1.00 (0.68–1.46)	1.00	0.91 (0.62–1.34)	0.63
Total score	0.45 (0.25–0.79)	0.006	0.56 (0.29–1.08)	0.08	0.54 (0.29–1.04)	0.07
FACIT-Sp						
Meaning/peace	0.56 (0.38–0.83)	0.004	0.70 (0.46–1.07)	0.10	0.64 (0.41–0.98)	0.040
Faith	0.85 (0.61–1.18)	0.32	0.82 (0.57–1.18)	0.29	0.75 (0.52–1.10)	0.14
Total score	0.59 (0.39–0.90)	0.013	0.70 (0.45–1.09)	0.12	0.62 (0.40–0.98)	0.042
Total score (G + Sp)	0.43 (0.25–0.77)	0.004	0.55 (0.29–1.04)	0.06	0.51 (0.27–0.96)	0.037

*HR* hazard ratio, *CI* confidence interval, *FACIT* functional assessment of cancer therapy, *FACIT-G* FACIT-General, *FACIT-Sp* FACIT-Spiritual. ^a^Disease progression was defined as either the first diagnosis of HCC or hepatitis-related death, such as hepatic failure and upper gastro-intestinal bleeding.^b^Using Cox proportional hazards models fitted to time-event data where event was either death associated with hepatitis or diagnosis of hepatocellular carcinoma. ^c^Adjusted for age and sex. ^d^Adjusted for age, sex, and baseline known risk factors^;^ cirrhosis, alanine transaminase (ALT), platelet count, alpha fetoprotein, diabetes, and alcohol-drinking. ^e^Adjusted for age, sex, baseline known risk factors, and treatment-related factors during the follow-up period^;^ ALT (most recent value) and results of antiviral treatments (sustained virological response, sustained biological response, or no response) as time-dependent variables. ^f^HR associated with a 1-point increment in the scores of the Stress Inventory scales and the FACIT scales

## Discussion

To our knowledge, this is the first longitudinal study to have examined the influence of psychosocial factors on the disease progression of patients with CHC. A behavioral pattern associated with the inhibition of emotional needs and a subdomain of QOL representing emotional wellbeing were found to be associated with the risk of either an onset of HCC or hepatitis-related death, independent of levels of known risk factors and treatment-related factors.

The development of HCC and/or hepatitis-related death are known to be predicted by the degree of fibrosis and inflammation, represented by cirrhosis, low platelet count, high AFP level, and high ALT level [32, 33]. As serum transaminase levels fluctuate in patients with CHC [34], a single measurement of ALT is insufficient to predict disease progression [35]. Indeed, in the present study, while the average of three recent measurements of ALT at entry did not predict subsequent events, a recent ALT level employed as a time-dependent variable in the Cox model did. We also attempted to introduce factors associated with antiviral treatments into analysis, by using time-dependent variables representing the effects of recent antiviral therapy, i.e. SVR, SBR, and no response. Other reported host factors include older age, male gender [32], overweight and diabetes [33], and high alcohol consumption [34]. We incorporated all of these known physical risk factors in the analysis.

Causality is crucial in discussing the association between psychosocial factors and physical conditions. Nagano et al. reported that a stronger tendency towards type-I behavior is associated with severer CHC [8]; however, as their study was cross-sectional, it could not exclude the possibility that psychosocial status was an effect, rather than a cause, of the physical conditions. In the present study, scores on both the SI type-I related scales and the FACIT subscales were more clearly associated with disease progression when patients who encountered an event or were censored within the first year of follow-up were excluded. This suggests that the sample contained patients whose psychosocial status was affected by impaired physical conditions, and that exclusion uncovered the effect of psychosocial factors on physical status. Further, while CHC severity’s association with physical, functional, and social/familial wellbeing was considerably attenuated by adjustment for baseline levels of known physical factors, its association with emotional wellbeing was not (see Table 4). Similarly, CHC severity’s association with unfulfilled need for acceptance was unchanged by adjustment for physical factors. This suggests that physical, functional, and social/familial wellbeing were influenced by physical status, and that the observed associations with disease progression were largely confounded by physical factors, while those with emotional wellbeing and unfulfilled need for acceptance were considerably independent of physical factors.

“Unfilled need for acceptance” refers to a chronic unfulfilled need for others’ acceptance [8]. This construct shares a common concept with repression of emotion, relevant constructs of which are associated with the progression of HIV infection [36, 37] and the development of malignant diseases [38, 39]. Additionally, unfilled need for acceptance is related to a lack of social support, which may also be important to the progression of HIV infection [40] and malignant diseases [41, 42]. QOL represents patients’ experience or perception of their degree of functioning in relation to illness, and reflects the effects of illness on multiple domains of patients’ health and ability to perform activities of daily life [43]. Regarding the subdomains of QOL assessed by the FACIT, scores on emotional wellbeing were most consistently associated with disease progression. By contrast, physical wellbeing’s association with disease progression was attenuated after controlling for physical factors, likely because this factor is more strongly affected by physical illness than emotional wellbeing (see Additional file 2: Table S2).

The mechanism underlying the relationship between psychosocial factors and CHC prognosis is complex. Improved wellbeing and behavioral patterns leading to wellbeing may accompany a healthier lifestyle (e.g. low alcohol consumption) [44]; however, they may also affect the liver-brain axis, thus exerting anti-viral and anti-carcinogenic effects on CHC via activation of the autonomic nervous system. Additionally, chronic stress is consistently associated with immune downregulation [45]. For example, wellbeing is associated with increased natural killer (NK) activity [46], while chronic psychosocial stress is associated with reduced NK activity [47–49]. The present data largely support these findings, as scores on the type-I SI scales and the FACIT scales were inversely and positively correlated with NK activity, respectively (see Additional file 3: Table S1 and Additional file 2: Table S2). Furthermore, decreased NK activity has been reported to be associated with elevated risk of developing HCC in cirrhotic patients [50]. Nonetheless, association between psychosocial factors and CHC prognosis in the present study did not change after controlling for baseline alcohol consumption and NK activity (cf. Table 3 and Additional file 4: Table S3; cf. Table 4 and Additional file 5: Table S4). This issue remains to be clarified.

About 60 % of the patients received antiviral therapy with IFN at least once by the end of follow-up. Because the antiviral therapy, especially when it results in SVR, is known to suppress the development of HCC [51, 52], we considered this factor in the analysis. Patients may receive the therapy on more than one occasion and their results can vary among SVR, SBR, and no response, so we addressed this issue by introducing time-dependent variables in the Cox proportional hazards model. In the model including the baseline known risk factors and a recent high value of ALT, SVR was not associated with disease progression as compared to no therapy. This may partly be explained by the fact that SVR occurred relatively later in the follow-up period (e.g., 43 % occurred 4 years or later from the start of follow-up). Thus, latent HCCs that developed in a potentially high-risk period preceding SVR may have become clinically detectable after SVR. Moreover, another time-dependent variable for a recent high value of ALT represents, at least in part, consequences of antiviral therapies. The adjustment for this factor, together with the above mentioned “disadvantageous” condition for SVR, might have masked the true effect of SVR in this particular cohort. Another issue to be discussed about antiviral therapy is IFN’s potential side effects on psychological status, including induction or progression of depression [53, 54]. It is possible that the IFN therapy might change the psychosocial status of some patients, and psychological and behavioral aspects as evaluated via the SI and FACIT scales might have been affected by antiviral therapy with IFN. We cannot address this issue as we only assessed the psychosocial factors at baseline, yet the observed associations with the psychosocial factors remained after controlling for the antiviral therapies that the patients received throughout the study period.

This study’s sample was small, which constitutes a major limitation. Despite the long follow-up time, the number of observed events was relatively small, and statistical power may have been insufficient to detect all associations between psychosocial factors and disease course. Although only the association with unfulfilled need for acceptance was statistically significant, the direction of disease course’ associations with the other type-I related scales of the SI seemed to be constant and consistent with previous reports [8], and may have been significant in a larger sample. Additionally, regarding the FACIT subscales, in the baseline and treatment-related factor adjusted models functional wellbeing and meaning/peace were marginally significantly associated and significantly associated with disease progression, respectively. As recently reported [43, 55], subjectively assessed functional wellbeing may be a prognostic factor for cancer patients, independent of objectively assessed performance status, which is a known prognostic factor for many malignant diseases [56, 57]. “Meaning/peace” represents an aspect of spirituality, a sense of meaning in one’s life, inner harmony, peacefulness, and a sense of comfort [58], and may be a critical resource for many patients in coping with chronic and/or malignant diseases [59]. Components of QOL such as functional wellbeing and meaning/peace should also be studied in larger samples. This study is also limited by the lack of collected data permitting the discussion of the biological mechanisms underlying the observed associations. We obtained a single measurement of NK activity at baseline; repeated measurements of NK activity or other factors representing the neuro-endocrino-immunological pathway would further advance understanding of the mechanisms underlying the associations observed in this study.

## Conclusion

This study is the first report a possible effect of psychosocial factors on the clinical course of patients with CHC, based on an 8-year prospective analysis that took into account known physical risk factors. Most possible predictive factors include a behavioral pattern relevant to the inhibition of emotional needs and a subdomain of QOL relevant to emotional wellbeing; however, the concept of type-I behavior and the other subdomains of QOL also warrant further examination in a larger sample of CHC patients. Regarding clinical practice, it may be more practical to address this topic if psychological interventions aimed at improving the QOL of patients with chronic diseases [60–62] are found to be able to reduce the risk of HCC and/or hepatitis-related death.

## Abbreviations

AFP, alpha-petoprotein; ALT, alanine transaminase; CHC, chronic hepatitis C; CI, confidence interval; CT, computed tomography; FACIT-Sp, Functional Assessment of Chronic Illnes Therapy-Spiritual; HCC, hepatocellular carcinoma; HCV, hepatitis C virus; HR, hazard ratio; IFN, interferon; NK, natural killer; QOL, quality of life; SBR, sustained biolochemical response; SI, stress inventory; SVR, sustained virological response; US, ultrasonography

## Additional files

Additional file 1: Table S1. Baseline associations between the Stress Inventory scales and physical factors. (DOCX 16 kb) Additional file 2: Table S2. Baseline associations between the FACIT scales and physical factors. (DOCX 16 kb) Additional file 3: Table S3. Psychosocial factors in association with subsequent disease progression^a^ in 240 patients with chronic hepatitis C: adjusted for baseline natural killer activity^b^. (DOCX 20 kb) Additional file 4: Table S4. Psychosocial factors in association with subsequent disease progression ^a^ in 227 patients with chronic hepatitis C, after excluding patients who met an event or was censored within the first 1 year of follow-up: adjusted for baseline natural killer activity ^b^. (DOCX 19 kb) Additional file 5. Assessment of biochemical measurements. (DOCX 14 kb)
