# Supplementary material for: Inhibition of emotional needs and emotional wellbeing predict disease progression of chronic hepatitis C patients: an 8-year prospective study
Source: Biopsychosoc Med. 2016 Jul 29;10:24. doi: 10.1186/s13030-016-0075-3 (PMC4966853; doi:10.1186/s13030-016-0075-3)
Supplement: Additional file 1: Table S1. — Baseline associations between the Stress Inventory scales and physical factors. (DOCX 16 kb) [file 13030_2016_75_MOESM1_ESM.docx]

**Additional file 1: Table S1.** Baseline associations between the Stress Inventory scales and physical factors

| Physical factors | Low sense of control | Object dependence of loss | | Unfulfilled needs for acceptance | | Altruism | | Type I score | |
| --- | --- | --- | --- | --- | --- | --- | --- | --- | --- |
| Age, years | －.05 | .10 |  | －.01 |  | －.08 |  | －.02 |  |
| Female sex | .05 | －.03 |  | －.15 |  | －.01 |  | －.08 |  |
| Duration, years | .07 | .01 |  | .05 |  | －.08 |  | .02 |  |
| Cirrhosis | －.06 | .03 |  | .05 |  | －.05 |  | －.01 |  |
| Alanine aminotransferase >= 40 IU/l | －.04 | －.00 |  | .03 |  | .03 |  | .01 |  |
| Platelet count < 100,000/mm^3^ | .12 | .13 | * | .19 | ** | .06 |  | .15 | * |
| Alpha fetoprotein >= 20 μg/l | .00 | .02 |  | .06 |  | .01 |  | .03 |  |
| Diabetes | .07 | .12 |  | .06 |  | .06 |  | .09 |  |
| Current alcohol-drinking | －.02 | －.07 |  | －.07 |  | －.15 | * | －.09 |  |
| Natural killer activity, % | －.10 | －.11 |  | －.17 | * | －.09 |  | －.15 | * |

Readings are Spearman’s rank correlation coefficients. *P < .05, **P < .01.
