# Supplementary material for: Inhibition of emotional needs and emotional wellbeing predict disease progression of chronic hepatitis C patients: an 8-year prospective study
Source: Biopsychosoc Med. 2016 Jul 29;10:24. doi: 10.1186/s13030-016-0075-3 (PMC4966853; doi:10.1186/s13030-016-0075-3)
Supplement: Additional file 2: Table S2. — Baseline associations between the FACIT scales and physical factors. (DOCX 16 kb) [file 13030_2016_75_MOESM2_ESM.docx]

**Additional file 2: Table S2.** Baseline associations between the FACIT scales and physical factors

| Physical factors | FACIT-G | | | | | | | | |
| --- | --- | --- | --- | --- | --- | --- | --- | --- | --- |
|  | Physical wellbeing | | Emotional wellbeing | | Functional wellbeing | | Social/familial wellbeing | Total score | |
| Age, years | .07 |  | －.08 |  | －.01 |  | .07 | .03 |  |
| Female sex | .02 |  | －.06 |  | .03 |  | .11 | .04 |  |
| Duration, years | .05 |  | .00 |  | .11 |  | .02 | .11 |  |
| Cirrhosis | －.24 | *** | －.12 |  | －.12 |  | .07 | －.11 |  |
| Alanine aminotransferase >= 40 IU/l | －.12 |  | －.02 |  | －.16 | * | －.03 | －.12 |  |
| Platelet count < 100,000/mm^3^ | －.22 | *** | －.18 | ** | －.14 | * | .01 | －.16 | * |
| Alpha fetoprotein >= 20 μg/l | －.18 | ** | －.11 |  | －.20 | ** | .08 | －.13 |  |
| Diabetes | .01 |  | .02 |  | .05 |  | .05 | .05 |  |
| Current alcohol-drinking | .13 |  | .15 | * | .21 | *** | .04 | .18 | ** |
| Natural killer activity, % | .20 | ** | .11 |  | .11 |  | －.06 | .13 | * |

FACIT, Functional Assessment of Cancer Therapy. Readings are Spearman’s rank correlation coefficients. *P < .05, **P < .01, ***P < .001.
