# Supplementary material for: Inhibition of emotional needs and emotional wellbeing predict disease progression of chronic hepatitis C patients: an 8-year prospective study
Source: Biopsychosoc Med. 2016 Jul 29;10:24. doi: 10.1186/s13030-016-0075-3 (PMC4966853; doi:10.1186/s13030-016-0075-3)
Supplement: Additional file 3: Table S3. — Psychosocial factors in association with subsequent disease progressiona in 240 patients with chronic hepatitis C: adjusted for baseline natural killer activityb. (DOCX 20 kb) [file 13030_2016_75_MOESM3_ESM.docx]

**Additional file 3: Table S3.** Psychosocial factors in association with subsequent disease progression^a^ in 240 patients with chronic hepatitis C: adjusted for baseline natural killer activity^b^

|  | Model 1^c^ | |  | Model 2^d^ | |
| --- | --- | --- | --- | --- | --- |
| Scale | HR (95% CI)^e^ | P value |  | HR (95% CI) | P value |
| **Stress Inventory** |  |  |  |  |  |
| Type-I-related scales |  |  |  |  |  |
| Low sense of control | 1.03 (0.80-1.32) | 0.82 |  | 1.02 (0.79-1.33) | 0.86 |
| Object dependence of loss | 1.16 (0.87-1.56) | 0.32 |  | 1.19 (0.87-1.61) | 0.28 |
| Unfulfilled need for acceptance | 1.16 (0.90-1.50) | 0.27 |  | 1.18 (0.91-1.53) | 0.22 |
| Altruism | 1.15 (0.86-1.55) | 0.35 |  | 1.10 (0.82-1.47) | 0.49 |
| Total score | 1.20 (0.85-1.69) | 0.31 |  | 1.19 (0.84-1.68) | 0.33 |
|  |  |  |  |  |  |
| **FACIT** |  |  |  |  |  |
| FACIT-G |  |  |  |  |  |
| Physical wellbeing | 0.87 (0.58-1.31) | 0.50 |  | 0.92 (0.61-1.40) | 0.71 |
| Emotional wellbeing | 0.72 (0.46-1.13) | 0.15 |  | 0.72 (0.45-1.14) | 0.16 |
| Functional wellbeing | 0.80 (0.56-1.14) | 0.22 |  | 0.81 (0.57-1.15) | 0.23 |
| Social/familial wellbeing | 0.98 (0.68-1.42) | 0.93 |  | 0.88 (0.61-1.26) | 0.48 |
| Total score | 0.69 (0.37-1.27) | 0.23 |  | 0.67 (0.37-1.22) | 0.19 |
| FACIT-Sp |  |  |  |  |  |
| Meaning/peace | 0.79 (0.53-1.18) | 0.24 |  | 0.70 (0.46-1.04) | 0.08 |
| Faith | 0.84 (0.60-1.19) | 0.33 |  | 0.75 (0.53-1.07) | 0.11 |
| Total score | 0.77 (0.51-1.18) | 0.23 |  | 0.67 (0.44-1.02) | 0.06 |
| Total score (G + Sp) | 0.67 (0.37-1.21) | 0.18 |  | 0.61 (0.34-1.09) | 0.10 |

HR: hazard ratio. CI: confidence interval. FACIT: Functional Assessment of Cancer Therapy. FACIT-G: FACIT-General. FACIT-Sp: FACIT-Spiritual. ^a^Disease progression was defined as either the first diagnosis of HCC or hepatitis-related death, such as hepatic failure and upper gastro-intestinal bleeding.^b^Using Cox proportional hazards models fitted to time-to-event data where event was either death associated with hepatitis or diagnosis of hepatocellular carcinoma. ^c^Adjusted for age, sex, and baseline known risk factors, i.e., cirrhosis, alanine transaminase (ALT), platelet count, alpha fetoprotein, diabetes, alcohol-drinking, and natural killer activity. ^d^Adjusted for age, sex, baseline known risk factors, and treatment-related factors during the follow-up period, i.e., ALT (most recent value) and results of antiviral treatments (sustained virological response, sustained biological response, or no response) as time-dependent variables. ^e^HR associated with a 1-point increment in the scores of the Stress Inventory scales and the FACIT scales.
