# Supplementary material for: Inhibition of emotional needs and emotional wellbeing predict disease progression of chronic hepatitis C patients: an 8-year prospective study
Source: Biopsychosoc Med. 2016 Jul 29;10:24. doi: 10.1186/s13030-016-0075-3 (PMC4966853; doi:10.1186/s13030-016-0075-3)
Supplement: Additional file 4: Table S4. — Psychosocial factors in association with subsequent disease progression a in 227 patients with chronic hepatitis C, after excluding patients who met an event or was censored within the first 1 year of follow-up: adjusted for baseline natural killer activity b. (DOCX 19 kb) [file 13030_2016_75_MOESM4_ESM.docx]

**Additional file 4: Table S4.** Psychosocial factors in association with subsequent disease progression ^a^ in 227 patients with chronic hepatitis C, after excluding patients who met an event or was censored within the first one year of follow-up: adjusted for baseline natural killer activity ^b^

|  | Model 1^c^ | |  | Model 2^d^ | |
| --- | --- | --- | --- | --- | --- |
| Scale | HR (95% CI)^e^ | P value |  | HR (95% CI) | P value |
| **Stress Inventory** |  |  |  |  |  |
| Type-I-related scales |  |  |  |  |  |
| Low sense of control | 1.12 (0.85-1.48) | 0.41 |  | 1.12 (0.84-1.49) | 0.44 |
| Object dependence of loss | 1.21 (0.89-1.67) | 0.23 |  | 1.24 (0.90-1.72) | 0.19 |
| Unfulfilled need for acceptance | 1.32 (1.00-1.74) | 0.049 |  | 1.34 (1.01-1.77) | 0.041 |
| Altruism | 1.22 (0.89-1.68) | 0.23 |  | 1.18 (0.86-1.61) | 0.32 |
| Total score | 1.37 (0.94-1.99) | 0.10 |  | 1.36 (0.94-1.99) | 0.11 |
|  |  |  |  |  |  |
| **FACIT** |  |  |  |  |  |
| FACIT-G |  |  |  |  |  |
| Physical wellbeing | 0.79 (0.51-1.24) | 0.31 |  | 0.84 (0.53-1.31) | 0.43 |
| Emotional wellbeing | 0.58 (0.36-0.94) | 0.028 |  | 0.58 (0.35-0.95) | 0.029 |
| Functional wellbeing | 0.72 (0.50-1.05) | 0.09 |  | 0.72 (0.49-1.05) | 0.09 |
| Social/familial wellbeing | 0.82 (0.56-1.18) | 0.28 |  | 0.86 (0.58-1.28) | 0.47 |
| Total score | 0.54 (0.28-1.06) | 0.07 |  | 0.54 (0.28-1.03) | 0.06 |
| FACIT-Sp |  |  |  |  |  |
| Meaning/peace | 0.66 (0.42-1.02) | 0.06 |  | 0.60 (0.38-0.93) | 0.023 |
| Faith | 0.82 (0.57-1.18) | 0.29 |  | 0.76 (0.52-1.10) | 0.15 |
| Total score | 0.66 (0.42-1.05) | 0.08 |  | 0.60 (0.38-0.95) | 0.029 |
| Total score (G + Sp) | 0.52 (0.27-1.00) | 0.05 |  | 0.49 (0.26-0.93) | 0.030 |

HR: hazard ratio. CI: confidence interval. FACIT: Functional Assessment of Cancer Therapy. FACIT-G: FACIT-General. FACIT-Sp: FACIT-Spiritual. ^a^Disease progression was defined as either the first diagnosis of HCC or hepatitis-related death, such as hepatic failure and upper gastro-intestinal bleeding. ^b^Using Cox proportional hazards models fitted to time-event data where event was either death associated with hepatitis or diagnosis of hepatocellular carcinoma. ^c^Adjusted for age, sex, and baseline known risk factors, i.e., cirrhosis, alanine transaminase (ALT), platelet count, alpha fetoprotein, diabetes, alcohol-drinking, and natural killer activity. ^d^Adjusted for age, sex, baseline known risk factors, and treatment-related factors during the follow-up period, i.e., ALT (most recent value) and results of antiviral treatments (sustained virological response, sustained biological response, or no response) as time-dependent variables. ^e^HR associated with a 1-point increment in the scores of the Stress Inventory scales and the FACIT scales.
