# Supplementary material for: Inhibition of emotional needs and emotional wellbeing predict disease progression of chronic hepatitis C patients: an 8-year prospective study
Source: Biopsychosoc Med. 2016 Jul 29;10:24. doi: 10.1186/s13030-016-0075-3 (PMC4966853; doi:10.1186/s13030-016-0075-3)
Supplement: Additional file 5. — Assessment of biochemical measurements. (DOCX 14 kb) [file 13030_2016_75_MOESM5_ESM.docx]

**Additional file 5: Assessment of biochemical measurements**

Venous blood samples were collected in citrate and serum tubes and spun at room temperature. All blood samples were frozen at -70°C until assay. The serogroup of HCV was determined using an enzyme immunoassay (ELISA) kit (Tonen Chemistry, Tokyo, Japan). Serum HCV RNA levels were measured with a quantitive HCV RNA PCR assay (Amplicor-M kit, Roche Diagnostic, Tokyo, Japan). Serum AFP levels and hyaluronic acid levels were evaluated using an ELISA kit (Diagnostic System Laboratories, Webster, TX) and a Latex Agglutination- Turbidimetric Immunoassay (Sujirebio Inc., Tokyo, Japan), respectively. Natural killer (NK) cell activity was measured using peripheral-blood mononuclear cells isolated on Ficoll-Hypaque gradients. Peripheral blood mononuclear cells were plated in 96 wells at four different effector to target ratios and coincubated with chromium-labeled K562 targets for 4 hours at 37°C。The cells were harvested, and the percentage of specific lysis was determined using the following formula: 100 (mean experimental counts per minutes cpm – mean spontaneous release cpm)/(mean maximum release cpm – mean spontaneous release cpm). The other biochemical data and the whole blood cell counts were measured by standard methods using a conventional automated analyzer.
